# Potential mammary carcinogens used in food contact articles: implications for policy, enforcement, and prevention

**DOI:** 10.3389/ftox.2024.1440331

**Published:** 2024-09-24

**Authors:** Lindsey V. Parkinson, Birgit Geueke, Jane Muncke

**Affiliations:** Food Packaging Forum Foundation, Zürich, Switzerland

**Keywords:** food packaging, food contact chemicals, breast cancer, hazard assessment, chemical safety, regulation

## Abstract

Many nations have food contact material (FCM) legislation purporting to protect citizens from hazardous chemicals, often specifically by regulating genotoxic carcinogens. Despite such regulations, cancers that are associated with harmful chemical exposures are highly prevalent, especially breast cancer. Using the novel Key Characteristics of Toxicants framework, Kay et al. found 921 substances that are potential mammary carcinogens. By comparing Kay et al.‘s chemicals list with our own Database on migrating and extractable food contact chemicals (FCCmigex), we found that 189 (21%) of the potential mammary carcinogens have been measured in FCMs. When limiting these results to migration studies published in 2020–2022, 76 potential mammary carcinogens have been detected to migrate from FCMs sold in markets across the globe, under realistic conditions of use. This implies that chronic exposure of the entire population to potential mammary carcinogens from FCMs is the norm and highlights an important, but currently underappreciated opportunity for prevention. Reducing population-wide exposure to potential mammary carcinogens can be achieved by science-based policy amendments addressing the assessment and management of food contact chemicals.

## Introduction

When travelling from source to table, foods contact a diverse array of food contact materials and articles (FCMs and FCAs), including processing equipment, packaging, and cookware. At each step along the way, substances from FCMs and FCAs, known as food contact chemicals (FCCs), can migrate into foodstuffs (Geueke et al., 2022). FCCs are regulated to ensure the safety of FCMs. United States regulations state that no (indirect) food additive, including FCCs, is considered safe if it causes cancer in humans or animals (Merrill, 1997). In the EU, materials must be manufactured so they do not transfer harmful constituents to food under normal use conditions at levels that are harmful to health (Regulation (EC) No 1935/2004) (European Commission, 2022; European Parliament and Council, 2004). Similar safety stipulations are applied by other governments and trade blocs globally (MERCOSUR, 1992; National People’s Congress, 2015). Regulatory agencies often require migration testing as part of FCM regulation and authorization, usually focused on restricting the use of cancer-causing genotoxic chemicals (Arvanitoyannis and Bosnea, 2004; Center for Food Safety and Applied Nutrition, 2021; Center for Food Safety and Applied Nutrition, 2007; Aguilar et al., 2008).

While global regulations and risk assessments of FCCs aim to prevent adverse health effects associated with FCC exposures, evidence suggests that these measures are not entirely effective. Several recent studies have identified links between FCCs and adverse health outcomes (Stevens et al., 2024; Trasande et al., 2024a; 2024b) or instances where the regulated allowable thresholds (reference dose, tolerable daily intake, etc.) are greater than the minimum observed adverse effect level in human populations (Maffini et al., 2021). Such examples indicate gaps in regulatory frameworks and assessment methodologies. Indeed, at least 127 known chemicals of concern (i.e., chemicals with hazard properties such as carcinogenic, mutagenic and toxic to reproduction (CMR), or endocrine disrupting chemicals (EDCs), etc.) have been shown to be present in FCMs, and 97 of these have evidence for migration (Zimmermann et al., 2022). Several other challenges exist for the risk assessment of food contact materials (Muncke et al., 2017), for example the lack of analytical methods and standards even for authorized food contact chemicals (Joint Research Centre, 2015) or robust exposure estimates (Alger et al., 2013). The European Parliament concluded that the implementation of EU FCM regulation is ineffective because it does not sufficiently protect human health (European Parliament, 2016). Similarly, the United States Government Accountability Office concluded in its investigation into the Food and Drug Administration’s (FDA) rules for FCMs that some FCCs may pose risks to human health (US Government Accountability Office, 2022).

To overcome these shortcomings, a generic approach to risk management (GRA) of chemicals, including FCCs, has been stipulated by the EU in its Chemicals Strategy for Sustainability (European Commission, 2020). GRA centers on identifying the intrinsic hazard properties of a substance, such as CMR, EDC, or environmental persistence. If a substance is identified as having such hazard properties of concern, restrictions or bans could be triggered without the need for extensively demonstrating human exposure.

The Key Characteristics of Toxicants (KC) framework can support GRA. KCs are inherent properties of chemical substances, derived from empirical evidence on how biological targets, such as biological molecules (DNA, proteins), cells, or tissues, are affected by types of chemicals with similar toxicity. For example, Smith et al. described the 10 KCs of carcinogens by identifying common molecular-level properties or interactions of known human carcinogens with biomolecules, like the induction of epigenetic alterations, causation of oxidative stress or chronic inflammation (Smith et al., 2016). This framework has also been expanded to other specific types of hazardous chemicals, such as endocrine disruptors (La Merrill et al., 2020), developmental toxicants (Arzuaga et al., 2019; Luderer et al., 2019), and immunotoxicants (Germolec et al., 2022). Recently, it has been suggested that the KC framework could also be used for predicting likely hazardous chemicals based on their molecular properties and interactions with biological molecular targets (Muncke et al., 2023).

Based on the KC framework, Kay et al. (2024) recently identified 921 substances with a high likelihood of contributing to breast cancer development, based on direct evidence of inducing mammary tumors in rodent models, genotoxicity testing, exhibiting endocrine disruption, or activation of other hormonal signaling pathways associated with the pathogenesis of breast cancer. In women, breast cancer is the most frequently diagnosed cancer and the globally leading cause of cancer deaths (Sung et al., 2021). As cancer is one of the few health effects specifically targeted in FCM regulation and testing, carcinogenic FCCs should be uncommon. Even so, chemical exposures, including to a few well-studied FCCs, have been linked to cancer development (Wan et al., 2022), covering the entire breast carcinogenesis process from tumor initiation (Wang et al., 2017) and growth (Koual et al., 2020), to metastasis (Koual et al., 2019) and resistance to chemotherapy (Lagunas-Rangel et al., 2022).

Here, we describe which potential mammary carcinogens identified by Kay et al. have been detected in FCMs and could contribute to human exposure because they have been shown to migrate into foodstuffs. Our findings imply that chronic exposure of the entire population to potential mammary carcinogens from FCMs is the norm and highlights an important, but currently underappreciated opportunity for prevention.

### Information sources for chemical comparisons

To compile their list of potential mammary carcinogens, Kay et al. (2024) used authoritative, publicly available datasets and identified 921 potential mammary carcinogens, of which 909 have CAS registry numbers.

The chemical substances intentionally used to manufacture FCMs, or that are present but have not been intentionally added (NIAS) in FCMs (e.g., contaminants, impurities of starting substances, reaction by-products), are known collectively as food contact chemicals (FCCs). There are at least 14,000 known FCCs (Geueke et al., 2022; Groh et al., 2021) but there may be as many as 100,000 potentially migrating FCCs (Grob et al., 2006; McCombie, 2018) when including all possible NIAS. However, identifying all NIAS is challenging and testing for individual compounds is impossible (Bradley and Coulier, 2007; Muncke et al., 2017; Oldring et al., 2023). The presence of chemicals in FCMs is investigated using two types of testing approaches: extraction or migration experiments. Extraction experiments employ conditions designed to maximize the release of all potentially migrating chemicals from the material and are considered a worst-case scenario. Migration experiments are designed to mimic real-use conditions as closely as possible, to provide an estimate of the likely human exposure to chemicals diffusing from these materials into foodstuffs.

The Database of migrating and extractable food contact chemicals (FCCmigex) (Food Packaging Forum Foundation, 2022; Geueke et al., 2022) is a systematic evidence map of 4,248 FCCs with CASRNs gathered from 1,312 publicly available studies and reports describing FCM migration and extraction experiments through October 2022.

Using the CASRNs, we identified FCCs known to be present in food contact materials and articles that are also included within the list of potential mammary carcinogens by Kay et al. (2024). List comparisons were made with python v3.11.5, and pandas v2.0.3.

### Potential breast carcinogens in FCMs

**FIGURE 1:**
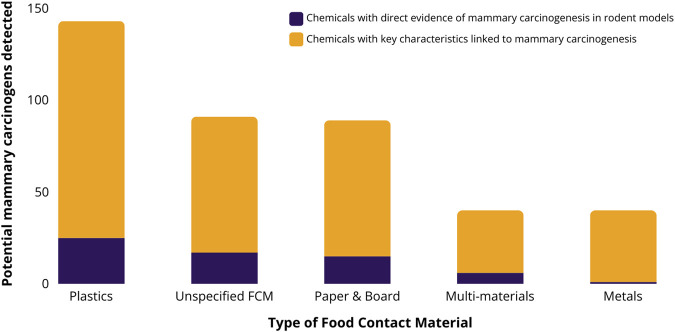
The number of potential mammary carcinogens as identified by Kay et al. (2024) that have been detected in migration or extraction studies of five food contact material groups. Food contact studies in FCCmigex were available online as of October 2022. Note: Potential mammary carcinogens detected, e.g., in coated cans or gaskets of metal closures, are assigned to the metal category. Columns are subdivided into food contact chemicals (FCCs) with direct evidence of carcinogenesis in rodent models (dark purple), and all other FCCs with key characteristics linked to breast carcinogenesis according to Kay et al. (yellow).

Of the 909 potential mammary carcinogens with CASRNs, 189 (21%) have been detected in FCMs (Excel Supplementary Table S1). Thirty of the chemicals have direct evidence of carcinogenesis in rodent models, another 67 are suspected to induce carcinogenesis based on their genotoxicity, and the remainder are highly likely or likely to be endocrine disruptors. Overall, 143 potential mammary carcinogens have been detected in plastic FCAs (76%), followed by non-specified materials (91; 48%), and paper and board (89; 47%), but all material groups except glass contained potential mammary carcinogens (Figure 1). When limiting results to FCCs migrating from FCMs into foods or food simulants, 121 potential mammary carcinogens have been detected.

By filtering for migration studies published within the last 3 years (2020, 2021, and 2022) for which there is information in FCCmigex (Food Packaging Forum Foundation, 2022) we captured a recent picture of likely human exposure to potential mammary carcinogens from FCMs via food ingestion.

Based on 181 migration studies from these 3 years, we identified 76 potential mammary carcinogens (Table 1). Of these, 61 (80%) have been measured to transfer from plastics, 23 (30%) from unspecified materials, 21 (28%) migrated from paper and board, 8 (11%) from metals, and 6 (8%) from multi-materials. Ten of the 76 chemicals detected in FCM migration studies in recent years have direct evidence of inducing mammary tumors in rodent models, e.g., benzene, styrene and several primary aromatic amines, and 35 are genotoxic (Kay et al., 2024) (Table 1, column “Key characteristic(s)”, values: mammary carcinogen “MC”, “Genotoxic”).

**TABLE 1:** Potential mammary carcinogens in food contact materials (FCMs) detected in migration experiments published in 2020, 2021, or 2022. Columns - FCCmigex: The number of database entries from FCCmigex related to migration studies from the years 2020–2022 compared to the total number of FCCmigex database entries where the chemical was detected in an FCM in studies published 1976–2022. FCMs studied: the FCMs tested in migration studies published in 2020–22 where the chemical was detected. Key characteristic(s): Evidence of selected KCs according to the criteria defined by Kay et al. (2024) that align with regulatory concerns in some jurisdictions, including directly inducing mammary carcinogenesis (MC) in rodent models, tested positive for genotoxicity (Genotoxic), high confidence or lower confidence endocrine disrupting (EDC+, EDC∼). EU REACH list(s): on the Community Rolling Action Plan (CoRAP) list, candidate substance of very high concern (Candidate list), or otherwise regulated. EU hazard class: Harmonized CLP classifications, known/presumed carcinogens and mutagens (1A/1B) marked in bold. Cells for which no relevant information was available are marked with (−).

CASRN	Name	FCCmigex migration entries Y2020-22/total entries Y1976-2022	FCMs studied, FCCmigex migration entries Y2020-22	Key characteristic(s) (Kay et al., 2024)	Inclusion EU REACH list(s)	EU hazard class (harmonized)
100-42-5	Styrene	13/99	Plastics, Paper & Board	MC, Genotoxic	-	Flam. Liq. 3 Repr. 2 Acute Tox. 4 STOT RE 1 Skin Irrit. 2 Eye Irrit. 2
100-51-6	Benzyl alcohol	1/11	Paper & Board	Genotoxic, EDC∼	CoRAP list	Acute Tox. 4
101-14-4	4,4′-Methylenebis(2-chloroaniline)	2/4	Plastics	MC, Genotoxic	Candidate list, Authorisation list (annex XIV)	**Carc. 1B** Acute Tox. 4 Aquatic Acute 1 Aquatic Chronic 1
101-77-9	4,4′-methylenedianiline	8/37	Multi-materials, Plastics, Unspecified	Genotoxic, EDC+	Candidate list, Authorisation list (annex XIV)	**Carc. 1B** Muta. 2 STOT SE 1 STOT RE 2 Skin Sens. 1 Aquatic Chronic 2
1034-01-1	Octyl gallate	1/2	Plastics	EDC+	-	Acute Tox. 4 Skin Sens. 1
103-41-3	Benzyl cinnamate	1/2	Plastics	EDC∼	-	No harmonized CLP classification
104-40-5	4-Nonylphenol	10/39	Plastics	EDC∼	Candidate list	No harmonized CLP classification
106-47-8	4-Chloroaniline	2/8	Plastics	Genotoxic, EDC+	-	**Carc. 1B** Acute Tox. 3 Skin Sens. 1 Aquatic Acute 1 Aquatic Chronic 1
108-39-4	m-Cresol	2/6	Plastics	Genotoxic, EDC∼	-	Acute Tox. 3 Skin Corr. 1B
111-71-7	Heptanal	1/19	Plastics	EDC∼	-	No harmonized CLP classification
112-05-0	Nonanoic acid	1/22	Paper & Board	EDC+	-	Aquatic Chronic 3 Eye Irrit. 2 Skin Irrit. 2
115-86-6	Triphenyl phosphate	2/25	Plastics, Unspecified	EDC+	CoRAP list	No harmonized CLP classification
117-81-7	Di(2-ethylhexyl) phthalate	44/329	Plastics, Unspecified, Metals, Multi-materials, Paper & Board	Genotoxic, EDC+	Candidate list, Authorisation list (annex XIV), Restriction list (annex XVII)	Repr. 1B
118-56-9	Homosalate	1/3	Unspecified	EDC+	-	No harmonized CLP classification
118-79-6	2,4,6-Tribromophenol	2/9	Plastics	Genotoxic, EDC+	CoRAP list	No harmonized CLP classification
118-82-1	4,4′-Methylenebis(2,6-di-t-butylphenol)	2/2	Plastics	EDC∼	CoRAP list	No harmonized CLP classification
119-61-9	Benzophenone	11/138	Unspecified, Plastics, Paper & Board	EDC∼	CoRAP list	No harmonized CLP classification
119-93-7	3,3′-Dimethylbenzidine	1/10	Plastics	MC, Genotoxic, EDC+	Restriction list (annex XVII)	**Carc. 1B** Acute Tox. 4 Aquatic Chronic 2
120-18-3	Naphthalene-2-sulfonic acid	1/1	Metals	EDC∼	-	No harmonized CLP classification
1207-12-1	4,6-Dimethyldibenzothiophene	1/5	Paper & Board	EDC∼	-	No harmonized CLP classification
121-33-5	Vanillin	2/20	Paper & Board, Unspecified	Genotoxic, EDC∼	-	No harmonized CLP classification
123-31-9	Hydroquinone	1/3	Paper & Board	Genotoxic, EDC+	CoRAP list	Aquatic Acute 1 Carc. 2 Muta. 2 Eye Dam. 1 Acute Tox. 4 Skin Sens. 1
124-22-1	1-Dodecanamine	1/1	Plastics	EDC+	-	No harmonized CLP classification
131-11-3	Dimethyl phthalate	9/66	Plastics, Unspecified	Genotoxic, EDC∼	-	No harmonized CLP classification
131-18-0	Dipentyl phthalate	4/13	Plastics	EDC∼	Candidate list	Repr. 1B Aquatic Acute 1
131-57-7	2-Hydroxy-4-methoxybenzophenone	4/8	Plastics	Genotoxic, EDC+	CoRAP list	No harmonized CLP classification
140-66-9	4-(1,1,3,3-tetramethylbutyl)phenol	4/24	Plastics, Unspecified	Genotoxic, EDC+	Candidate list	Skin Irrit. 2 Eye Dam. 1 Aquatic Acute 1 Aquatic Chronic 1
143-08-8	1-Nonanol	2/7	Plastics, Unspecified	EDC∼	-	No harmonized CLP classification
1478-61-1	Bisphenol AF	4/14	Plastics	EDC+	-	No harmonized CLP classification
14938-35-3	4-Pentylphenol	3/3	Plastics	EDC+	-	No harmonized CLP classification
1638-22-8	4-Butylphenol	2/2	Plastics	EDC+	-	No harmonized CLP classification
1987-50-4	4-Heptylphenol	3/3	Plastics	EDC+	Candidate list	No harmonized CLP classification
21245-02-3	2-Ethylhexyl 4-(dimethylamino)benzoate	6/54	Unspecified, Paper & Board	EDC∼	-	No harmonized CLP classification
23128-74-7	Irganox 1098	3/5	Plastics	EDC+	-	No harmonized CLP classification
2440-22-4	2-(2H-Benzotriazol-2-yl)-4-methylphenol	1/10	Plastics	EDC∼	CoRAP list	No harmonized CLP classification
24602-86-6	Tridemorph	1/1	Paper & Board	EDC+	-	Acute Tox. 4 Skin Irrit. 2 Aquatic Acute 1 Aquatic Chronic 1 Repr. 1B
24851-98-7	Methyl dihydrojasmonate	1/5	Plastics	EDC+	-	No harmonized CLP classification
26172-55-4	5-Chloro-2-methyl-3(2H)-isothiazolone	1/7	Paper & Board	Genotoxic, EDC+	-	No harmonized CLP classification
2772-45-4	2,4-Bis(1-methyl-1-phenylethyl)phenol	1/4	Plastics	EDC∼	-	No harmonized CLP classification
42978-66-5	Tripropylene glycol diacrylate	1/10	Plastics	EDC∼	-	Eye Irrit. 2 Skin Irrit. 2 Skin Sens. 1 STOT SE 3 Aquatic Chronic 2
4376-20-9	Mono-(2-ethylhexyl) phthalate	2/7	Plastics	EDC∼	-	No harmonized CLP classification
499-75-2	5-Isopropyl-2-methylphenol	2/72	Unspecified	Genotoxic, EDC∼	-	No harmonized CLP classification
57-11-4	Octadecanoic acid	8/53	Paper & Board, Plastics, Unspecified	EDC+	-	No harmonized CLP classification
5888-33-5	Isobornyl acrylate	1/1	Plastics	EDC∼	-	Skin Sens. 1A
599-64-4	4-Cumylphenol	1/1	Plastics	EDC+	-	No harmonized CLP classification
620-92-8	Bisphenol F	10/23	Metals, Plastics	EDC∼	-	No harmonized CLP classification
645-56-7	4-Propylphenol	3/3	Plastics	EDC∼	-	No harmonized CLP classification
6683-19-8	Irganox 1010	7/66	Plastics	EDC+	-	No harmonized CLP classification
678-39-7	8:2 FTOH	2/15	Unspecified	EDC∼	-	No harmonized CLP classification
68-26-8	Retinol	1/1	Unspecified	Genotoxic, EDC∼	-	No harmonized CLP classification
6846-50-0	2,2,4-Trimethyl-1,3-pentanediol diisobutyrate	4/32	Unspecified, Plastics, Paper & Board	EDC∼	-	No harmonized CLP classification
71-43-2	Benzene	5/29	Plastics, Paper & Board	MC, Genotoxic	Restriction list (annex XVII)	**Carc. 1A** **Muta. 1B** Flam. Liq. 2 STOT RE 1 Asp. Tox. 1 Eye Irrit. 2 Skin Irrit. 2
77-40-7	Bisphenol B	3/4	Plastics	EDC+	Candidate list	No harmonized CLP classification
78-63-7	2,5-Dimethyl-2,5-di-(tert-butylperoxy)hexane	2/2	Metals, Paper & Board	EDC∼	-	No harmonized CLP classification
79-94-7	Tetrabromobisphenol A	2/13	Plastics	EDC+	CoRAP list, Candidate list	Aquatic Acute 1 Aquatic Chronic 1
79-97-0	Bisphenol C	3/4	Metals, Plastics	Genotoxic, EDC∼	CoRAP list	No harmonized CLP classification
80-05-7	Bisphenol A	42/253	Metals, Unspecified, Plastics, Paper & Board	Genotoxic, EDC+	Candidate list, CoRAP list, Restriction list (annex XVII)	Repr. 2 STOT SE 3 Eye Dam. 1 Skin Sens. 1
80-09-1	4,4′-Sulfonyldiphenol	7/26	Plastics, Paper & Board	Genotoxic, EDC+	CoRAP list	No harmonized CLP classification
842-0-9	C.I. Solvent Yellow 14	1/1	Plastics, Paper & Board	Genotoxic, EDC+	-	Muta. 2 Carc. 2 Skin Sens. 1 Aquatic Chronic 4
84-61-7	Dicyclohexyl phthalate	4/34	Plastics, Unspecified	EDC∼	Candidate list, CoRAP list	No harmonized CLP classification
84-66-2	Diethyl phthalate	12/133	Paper & Board, Plastics, Unspecified	Genotoxic, EDC∼	CoRAP list	No harmonized CLP classification
84-69-5	Diisobutyl phthalate	22/197	Plastics, Unspecified, Metals, Multi-materials, Paper & Board	EDC∼	Candidate list, Authorisation list (annex XIV)	Repr. 1B
84-74-2	Dibutyl phthalate	39/290	Plastics	Genotoxic, EDC∼	Candidate list, Authorisation list (annex XIV), Restriction list (annex XVII)	Repr. 1B Aquatic Acute 1
85-68-7	Benzyl butyl phthalate	18/99	Metals, Paper & Board, Plastics, Unspecified	Genotoxic, EDC+	Candidate list, Authorisation list (annex XIV), Restriction list (annex XVII)	Repr. 1B Aquatic Acute 1 Aquatic Chronic 1
87-62-7	2,6-Dimethylaniline	2/8	Multi-materials, Plastics	Genotoxic, EDC∼	-	Carc. 2 Acute Tox. 4 Skin Irrit. 2 STOT SE 3 Aquatic Chronic 2
89-83-8	Thymol	2/4	Unspecified	Genotoxic, EDC∼	-	Acute Tox. 4 Aquatic Chronic 2 Skin Corr. 1B
91-59-8	2-Naphthylamine	2/5	Plastics	EDC+	Restriction list (annex XVII)	**Carc. 1A** Acute Tox. 4 Aquatic Chronic 2
91-94-1	3,3′-Dichlorobenzidine	4/6	Multi-materials, Plastics	MC, Genotoxic	Restriction list (annex XVII)	**Carc. 1B** Acute Tox. 4 Skin Sens. 1 Aquatic Acute 1 Aquatic Chronic 1
92-67-1	4-Biphenylamine	4/8	Plastics	MC, Genotoxic	Candidate list, Restriction list (annex XVII)	**Carc. 1A** Acute Tox. 4
92-87-5	Benzidine	6/11	Plastics	MC, Genotoxic, EDC+	Restriction list (annex XVII)	**Carc. 1A** Acute Tox. 4 Aquatic Acute 1 Aquatic Chronic 1
94-13-3	Propylparaben	1/4	Plastics	EDC+	CoRAP list	No harmonized CLP classification
94-26-8	4-Hydroxybenzoic acid butyl ester	1/1	Plastics	EDC+	Candidate list	No harmonized CLP classification
95-53-4	2-Methylaniline	2/12	Plastics, Unspecified	MC, Genotoxic	Candidate list	**Carc. 1B** Acute Tox. 3 Eye Irrit. 2 Aquatic Acute 1
95-69-2	4-Chloro-2-methylaniline	3/7	Plastics, Unspecified	Genotoxic, EDC+	-	**Carc. 1B** Muta. 2 Acute Tox. 3 Aquatic Acute 1 Aquatic Chronic 1
95-80-7	2,4-Diaminotoluene	4/22	Multi-materials, Plastics	MC, Genotoxic, EDC∼	Candidate list	**Carc. 1B** Muta. 2 Repr. 2 Acute Tox. 3 Acute Tox. 4 STOT RE 2 Skin Sens. 1 Aquatic Chronic 2
97-56-3	o-Aminoazotoluene	2/4	Plastics	MC, Genotoxic, EDC+	Candidate list	**Carc. 1B** Skin Sens. 1

At least 40 of the 76 potential mammary carcinogens detected in the recent migration studies have already been classified with some sort of hazard warning by at least one regulatory agency. For example, 19 and six potential mammary carcinogens are already included in the EU REACH Candidate and Authorisation lists, respectively (European Parliament and Council, 2006). Thirteen chemicals are classified as class-1 carcinogen or mutagen according to Annex VI of the CLP Regulation (European Parliament and Council, 2008). Proposition 65 in California lists 23 of the suspected mammary carcinogens while South Korea’s hazardous chemicals list contains 22, and the Rotterdam Convention lists nine (National Institute of Environmental Research, 2024; OEHHA, 2024; Wagner et al., 2024). At least 26 potential mammary carcinogens detected in recent migration studies belong to categories currently under regulatory scrutiny at various agencies, including six bisphenols, seven ortho-phthalates, twelve aromatic amines, and one PFAS (Lambré et al., 2023; Washington State Department of Ecology, 2023; European Parliament and Council, 2004; US FDA, 2024b).

The ten FCCs with direct evidence of mammary carcinogenesis in rodent models were recently detected in migration studies from FCMs purchased in multiple countries in the EU (Germany, Hungary, Poland, Spain), as well as the United Kingdom, United States, China, and Malaysia.

In all, the 76 recently detected potential mammary carcinogens were in FCMs purchased from markets all over the world including the United States of America (e.g., Sapozhnikova and Nuñez, 2022; Taylor and Sapozhnikova, 2022), India (e.g., Chapke et al., 2022; Mukhopadhyay et al., 2022), China (e.g., Han et al., 2021; Luo et al., 2022), Nigeria (e.g., Ibeto et al., 2022; Ucheana et al., 2022), Ghana (Angnunavuri et al., 2022; Ayamba et al., 2020), Spain (e.g., Blanco-Zubiaguirre et al., 2021; Song et al., 2022), Mexico (de Anda-Flores et al., 2021), Austria (Banaderakhshan et al., 2022), Canada (Siddique et al., 2021; Xu et al., 2023), Syria (Wissam, 2021), Poland (Marć, 2020), Iran (Cheshmazar et al., 2021), Malaysia (Naziruddin et al., 2021; Naziruddin et al., 2020), Denmark (Tisler and Christensen, 2022; Tsochatzis et al., 2021), Egypt (Gamil et al., 2022), Turkey (Alp and Yerlikaya, 2020), Greece (Kalogiouri et al., 2021), and Brazil (Oliveira et al., 2020).

## Discussion

The presence of these confirmed and potential mammary carcinogens, despite regulation since 1958 in the United States (US Congress, 1958) and 1976 in the EU (European Council, 1976) specifically targeting carcinogens in FCMs, highlights the shortcomings and gaps of the current regulatory system.

Of the ten recently-detected, migrating FCCs with direct evidence of mammary carcinogenesis, styrene is particularly illustrative. Styrene is a high production volume chemical with nearly 4 decades of evidence of migration into foods that continues to be allowed in FCMs (European Commission, 2024; US FDA, 2024a) despite its classification in the EU as a suspected reproductive toxicant in 2012 (ECHA, 2013), listing as a carcinogen according to California Proposition 65 in 2016 (OEHHA, 2014), and addition to the Republic of Korea’s list of toxic substances in 2021 (National Institute of Environmental Research, 2024). Additionally, styrene was highlighted as an FCC of concern that does not fit the goals outlined in the Chemicals Strategy for Sustainability in 2021 and 2022 (Groh et al., 2021; Zimmermann et al., 2022), as well the recent inclusion in the PlastChem red list and in Kay et al.‘s potential mammary carcinogens list (Kay et al., 2024; Wagner et al., 2024).

In the EU specifically, REACH (Regulation (EC) No 1907/2006) mandates the substitution of SVHCs in industrial products and consumer articles based on their intrinsic hazard properties (European Parliament and Council, 2006). However, verifying that a substance has an intrinsic hazard property, or even multiple of such properties, does not automatically translate to restrictions in FCMs (Geueke and Muncke, 2018; Zimmermann et al., 2022). This is clearly illustrated by the 19 SVHCs included in the list of potential mammary carcinogens that have been recently detected in migration studies of FCAs on the EU market. Such instances beg the question whether the current FCM regulatory system based on risk assessment creates a false sense of safety.

Mammary carcinogenicity is only one endpoint. As consensus around KCs of other health effects develops over the coming years (see www.keycharacteristics.org), the extent to which the population is being exposed to hazardous chemicals via everyday products will become clearer. Regulatory mechanisms should be in place to quickly respond to health concerns that the growing evidence brings to light.

It is important to note that a GRA-based system without consideration of alternatives could lead to regrettable substitutions (Barlow et al., 2015). A balance between protecting public health and the environment, and maintaining technological and economic viability, will need to be found. The KC framework can help with this by identifying intrinsic chemical properties in the context of biological systems that are predictive of hazard. It also simplifies identification of hazardous chemicals by eliminating the need to define each chemical’s adverse outcome pathway or critical mode of action (Guyton et al., 2018), while enabling the identification and prioritization of chemicals based on a wide range of hazardous traits (Luderer et al., 2019). With clear indicators of harmful characteristics, and how any chemical with those characteristics will be regulated, industries can be encouraged to innovate and develop safer alternatives using the KC framework.

A hazard-based regulation using GRA helps to protect against unforeseen exposures and risks that may not be adequately captured by current risk assessment methodologies and risk management approaches (e.g., Maffini et al., 2021). It acknowledges that exposure scenarios can change over time with new uses, technologies, and environmental pathways, offering a safety net against unexpected threats. Simplified chemical assessments relying on a robust, evidence-based component, such as laid out in the KC framework, streamline the assessment process, so that it can be standardized and undertaken by any regulatory agency worldwide, with minimal input data, short time requirements, and at reasonably low cost.

Moving forward, it is advisable for regulatory bodies to reconsider the integration of GRA and hazard-based criteria into the regulation of FCMs, as mandated in the EU by its Chemicals Strategy for Sustainability (European Commission, 2020). This entails not only identifying substances of concern but also addressing the broader categories of chemicals with the potential for diverse adverse health outcomes. Incorporating the KCs of chemicals that induce breast carcinogenesis via different mechanisms of action, of which genotoxicity is only one, into regulation may allow regulators to live up to the ideals espoused in FCM regulations.

Additionally, fostering international collaboration and harmonization of regulatory standards could further strengthen global efforts to manage the risks associated with chemical migration from FCMs. Such proactive measures are crucial in navigating the complexities of the modern food supply chain and ensuring the safety and wellbeing of consumers worldwide–today and for future generations.

## Conclusion

While the transition to the generic approach to risk management (GRA), effectively a hazard-based regulatory framework, represents a significant shift in the approach to FCM regulation worldwide, we argue that it is an important and underappreciated opportunity for prevention. Even when considering only a single health endpoint, mammary carcinogenicity, and recent FCM migration data, there are at least 76 known or potential mammary carcinogens migrating from FCMs across the global market. This finding implies that public health protection can be significantly improved by modernized FCM regulations with a focus on hazard identification, for example by employing the KC framework.

## Data Availability

Publicly available datasets were analyzed in this study. This data can be found here: Kay et al. 2024 (https://ehp.niehs.nih.gov/doi/10.1289/EHP13233#supplementary-materials) and FCCmigex (https://www.foodpackagingforum.org/fccmigex).
